# *Netlang*: A software for the linguistic analysis of corpora by means of complex networks

**DOI:** 10.1371/journal.pone.0181341

**Published:** 2017-08-23

**Authors:** Lluís Barceló-Coblijn, Diego Serna Salazar, Gustavo Isaza, Luis F. Castillo Ossa, Manuel G. Bedia

**Affiliations:** 1 Department of Catalan Philology and General Linguistics, University of the Balearic Islands, Palma, Balearic Islands, Spain; 2 Departamento de Sistemas e Informatica, Universidad de Caldas, Manizales, Caldas, Colombia; 3 Departamento de Ingeniería Industrial Universidad Nacional de Colombia sede Manizales, Caldas, Colombia; 4 Departamento de Sistemas e Informatica, Universidad de Caldas, Manizales, Caldas, Colombia; 5 Department of Computer Science and Systems Engineering, University of Zaragoza, Zaragoza, Aragón, Spain; Universitat Rovira i Virgili, SPAIN

## Abstract

To date there is no software that directly connects the linguistic analysis of a conversation to a network program. Networks programs are able to extract statistical information from data basis with information about systems of interacting elements. Language has also been conceived and studied as a complex system. However, most proposals do not analyze language according to linguistic theory, but use instead computational systems that should save time at the price of leaving aside many crucial aspects for linguistic theory. Some approaches to network studies on language do apply precise linguistic analyses, made by a linguist. The problem until now has been the lack of interface between the analysis of a sentence and its integration into the network that could be managed by a linguist and that could save the analysis of any language. Previous works have used old software that was not created for these purposes and that often produced problems with some idiosyncrasies of the target language. The desired interface should be able to deal with the syntactic peculiarities of a particular language, the options of linguistic theory preferred by the user and the preservation of morpho-syntactic information (lexical categories and syntactic relations between items). *Netlang* is the first program able to do that. Recently, a new kind of linguistic analysis has been developed, which is able to extract a complexity pattern from the speaker's linguistic production which is depicted as a network where words are inside nodes, and these nodes connect each other by means of edges or links (the information inside the edge can be syntactic, semantic, etc.). The *Netlang* software has become the bridge between rough linguistic data and the network program. *Netlang* has integrated and improved the functions of programs used in the past, namely the DGA annotator and two scripts (*ToXML*.*pl* and *Xml2Pairs*.*py*) used for transforming and pruning data. *Netlang* allows the researcher to make accurate linguistic analysis by means of syntactic dependency relations between words, while tracking record of the nature of such syntactic relationships (subject, object, etc). The *Netlang* software is presented as a new tool that solve many problems detected in the past. The most important improvement is that *Netlang* integrates three past applications into one program, and is able to produce a series of file formats that can be read by a network program. Through the *Netlang* software, the linguistic network analysis based on syntactic analyses, characterized for its low cost and the completely non-invasive procedure aims to evolve into a sufficiently fine grained tool for clinical diagnosis in potential cases of language disorders.

## Introduction

The study of the language capacity–and the potential linguistic disorders a speaker can develop–experienced a great evolution when the first brain areas related to language were detected (i.e., Broca's, Wernicke's area [1]). Subsequent work, have proven that the initial model was in fact too simple (e.g, [2,3]). Language development has been a really contentious issue: Chomsky’s claim that domain-general learning is unable to account for language acquisition (cf. i.a. [4]) has never been completely accepted. While Chomsky advocates for some innate knowledge of grammar, authors like Mehler ([5], et seq.) have hypothesized that learning a language amounts to “unlearning” others (cf. Also [6, 7, 8] among many others). Instead, for Tomasello ([9] et seq.) syntactic structures and categories are learned “one after another”.

As a consequence of these debates, studies on atypical development have also been affected by different viewpoints. For the study of language development, the analysis of linguistic production in clinical linguistics has become a cross-disciplinary field frequented by linguists, psycholinguists and psychologists in particular. For example, [10] have analyzed the structural characteristics of syntactic constructions produced by speakers with Williams syndrome; analysis of linguistic production of speakers with Down syndrome has been deeply explored in the last decades (cf. among many others [11,12]); while comparisons between the linguistic production of different clinical population groups are also of great interest and an important source of information [13,14].

The analysis of language is useful for finding markers that could account for language development during ontogeny, being typical, atypical, bilingual, multilingual, etc. Several approaches have tried to offer indicators able to extract statistical information from the linguistic source, using sometimes simple indicators and sometimes more complex software for syntactic analyses e.g., counting the number words; or the *Index of Productive Syntax* [15] focused in reading sentences, repeating sentences or other expressions; or the Mean Length of Utterance (MLU [16]), which consists in taking randomly 100 sentences produced by the speaker, then counting their words, and finally dividing the number of words by 100 [17]. MLU provides a numeric indicator and if the speakers fall between 4 and 5, it is considered a typical index of linguistic production; other versions of the MLU take into account different numbers (e.g., 50 instead of 100) or the 5 longest sentences, or take the morpheme–not the word–as unit.

When it is so evident that the linguistic production is not the typical one, clinical linguists descend to the analysis of sentences and utterances. These analyses, depending on their accuracy, can uncover several aspects that go beyond word counting. For example, experts can observe problems in the use of pronouns, or in the production of embedded sentences, or problems in the correct use of verbal morphology can be observed (e.g., [13]). Both MLU and linguistic analysis of sentences are able to point clinical aspects of speakers’ linguistic production. While MLU is an indicator that must be taken into account along with other indicators (e.g., IQ, mental age, etc.), sometimes linguistic analyses try by themselves to be so accurate that can discriminate between clinical conditions. Nonetheless, MLU sometimes lacks accuracy and has been considered as an imperfect proxy for syntactic development due to the well-known problems of the variability in morphological structure among languages (words may entail just one or several morphemes), and the important role of functional words for syntax acquisition [18,19].

The analysis of language can be improved by computer tools that connect these analyses with powerful programs able to detect the pattern of language complexity of an individual. The use of computational techniques in clinical studies of language related abilities is an emergent approach currently highly topical. Thus, [20] argue that automated speech analysis allows the measurement of ‘subtle, clinically relevant mental state changes in emergent psychosis’ which cannot be detected by clinicians without the help of these methods, concluding that ‘recent developments in computer science, including natural language processing, could provide the foundation for future development of objective clinical tests for psychiatry. Hence, the analysis of linguistic production has become crucial and computer tools are now a key tool for scholars in this field. Most analysis have until now focused on words, sentences or phrases considered as single units. There exist several automatic systems for language analysis that are useful at different levels and with different levels of accuracy regarding linguistic theory [21]. Although we think that automatic analysis is desirable and hope that eventually this will be the most common methodology, one of the problems of current research on language acquisition is that such systems are not always suitable for cross-linguistic studies, or for approaches that are committed to a particular school of linguistics. We therefore combine manual analyses with computational tools that help in extracting additional information from those analyses. In linguistics the conception of the computational system that would sustain the capability for language largely lays on the conception of grammar (and language) one has. Nevertheless, it is possible to create a linguistic tool which is both structured according to a linguistic type of grammar and open enough to be customized to include theoretical developments and novelties (or typological particularities of a language).

Scarborough’s IPS addresses some of the weaknesses in the MLU [15,16]. The IPS score is obtained from a corpus of 100 utterances, within which 56 specific language structures can be found. By combining the IPS with NLP techniques, [22] have presented a new tool which represents a substantial evolution of the already classic IPS. From our perspective, the most important aspect of their approach is the introduction of dependency structures [23] for the identification of grammatical relationships.

Generally speaking, past methodologies aimed to capture in one way or another, the level of syntactic complexity reached by the speaker. Next, we present a technique which is able to capture the complexity of the speaker's ability to syntactically combine lexical items. Our specific approach follows the hypothesis that Syntactically Analyzed Networks (SAN) built from precise syntactic analyses made by a linguist can be used as endophenotypes [24]: biological markers uniting genotype and phenotype. SAN is a relatively recent technique able to extract patterns of speech by combining manual syntactic analyses with network analysis (e.g. [25, 26]). It consists of the next stages: from a sample of speech every linguistic expression produced by the speaker is syntactically analyzed, indicating how words syntactically depend on each other, following Dependency Grammar. Previous work has in fact suffered from experimental scripts and software that was old and not specific to this kind of approach. The novelty of the present work is that we have developed specific software that solves many of the problems of previous one. Some classical deficits related with preservation of linguistic information and linguistic analysis have been finally overcome thanks to the *Netlang* software, a new tool for the manual syntactic analysis of corpora that is useful for the syntactic annotation and at the same time does not preclude the analysis of typologically different languages which could greatly differ in the composition of lexical units.

## 1. The studies of complexity and language

From a physics perspective, the study of linguistic complexity has also been addressed. With the advent of modern network studies, particularly fuelled by [27], the application of networks to complex systems has reached the province of linguistics too. As a matter of fact, the application of networks to linguistic studies is not brand new, as it has been previously applied to phonology–clearly the linguistic area that has gotten most attention–cf. among many others, [28,29]. Moreover, the applicability of these types of approaches is also being exported to the clinical studies [30]. On another linguistic front, adopting a word co-occurrence approach, [31] analyzed the semantic network growth in typical and late talkers and their results seemed to support the view that small-world connectivity and lexical development are somehow linked in individual children. [32] have shown that the distribution of co-occurrences of words reflect a small-world network pattern. However, co-occurrence has also been called into question due to its apparent soft connection to linguistic theory [33]. [34] suggests that “word-adjacency networks should not be represented as networks until a convincing network process using it in a meaningful and easily describable way is defined”. A rather different approach was adopted by [25] who studied the linguistic development of children acquiring English, by combining syntactic analysis with network representation. Let’s call Syntactically Analyzed Networks (SAN) those networks created on the basis of linguistic corpora that have been syntactically analyzed line by line by a human. Their results show that healthy children follow a particular schema of language development, characterized by a combination of linear and non-linear progress. This work was replicated by [26] who analyzed German, Dutch and Spanish children and their results showed that they developed their linguistic capability in three different phases, each well depicted by a kind of network: tree-like network, scale-free network and small-world network. Finally, [35] have expanded the analysis of linguistic production by means of SANs to Catalan, French, Italian and Basque in healthy developmental conditions, and to a large group of speakers affected by Down syndrome. Their results clearly show that DS speakers produce a very different kind of network and that their linguistic development is not delayed, but it simply follows a divergent developmental path. Until now, this technique based on SANs, although attractive to practitioners interested in obtaining more and better information about their patients' linguistic capability, has been too complex and problematic to be adopted as a daily procedure. To be something adopted in clinical linguistic approaches, there should not be problems with linguistic idiosyncrasies or with scrip (see section 2). The tools should be more accessible and, if possible, open enough to be applied to any language. For this reason, during the years 2014–15 a new software was developed in order to overcome those problems, with the intention of offering a computer tool that could be used by non-linguists (although the more fine-grained information is desired, the higher the level of knowledge on linguistics will be required).

In the next sections the software will be put to the test showing the improvements for the application of this kind of analyses. After solving the most important pitfalls of the procedure, a series of linguistic corpora have been syntactically analyzed with *Netlang*. Then, the output files have been analyzed with a network program showing that this procedure is interesting for tracking the development of a typical child, a child with neurological impairment affecting language, and also for studies on bilingualism.

## 2. Software design and implementation

Four recent studies [24, 25, 26, 35] followed the protocol developed by [36] including a set of computer tools rather experimental that were not immune to several unforeseen difficulties.

The linguistic network approach consists basically of two phases:

1. linguistic analysis of utterances by means of dependency relations between words (or morphemes),
2. conflation of these structured utterances into a network.

Phase (1) strongly relied upon two scripts and one annotation program: a script (*ToXML*.*pl*.) was used in order to prune the data and give the suitable format to the output file. Due to the fact that the texts were taken from the folders with (clinical) linguistic information from CHILDES [42], the format of.*cha* files had to be removed. The output was an.*xml* file with the required format by the Dependency Grammar Annotator (DGA), that allowed the syntactic analysis of sentences. Once the text file was ready, the human linguist needed the program for linguistic analysis *Dependency Grammar Annotator or DGA* [37] and the text was analyzed. Once the corpora was syntactically annotated, *DGA*'s output was a.*xml* file that could not be read by a network program. Hence, a script *Xml2Pairs*.*py* was applied for transforming the syntactic analysis into a file readable by a network program. *Xml2Pairs*.*py* is a script able to transform the relationships between words within a sentence in ordered columns, a format accepted by network programs.

**Fig 1:**
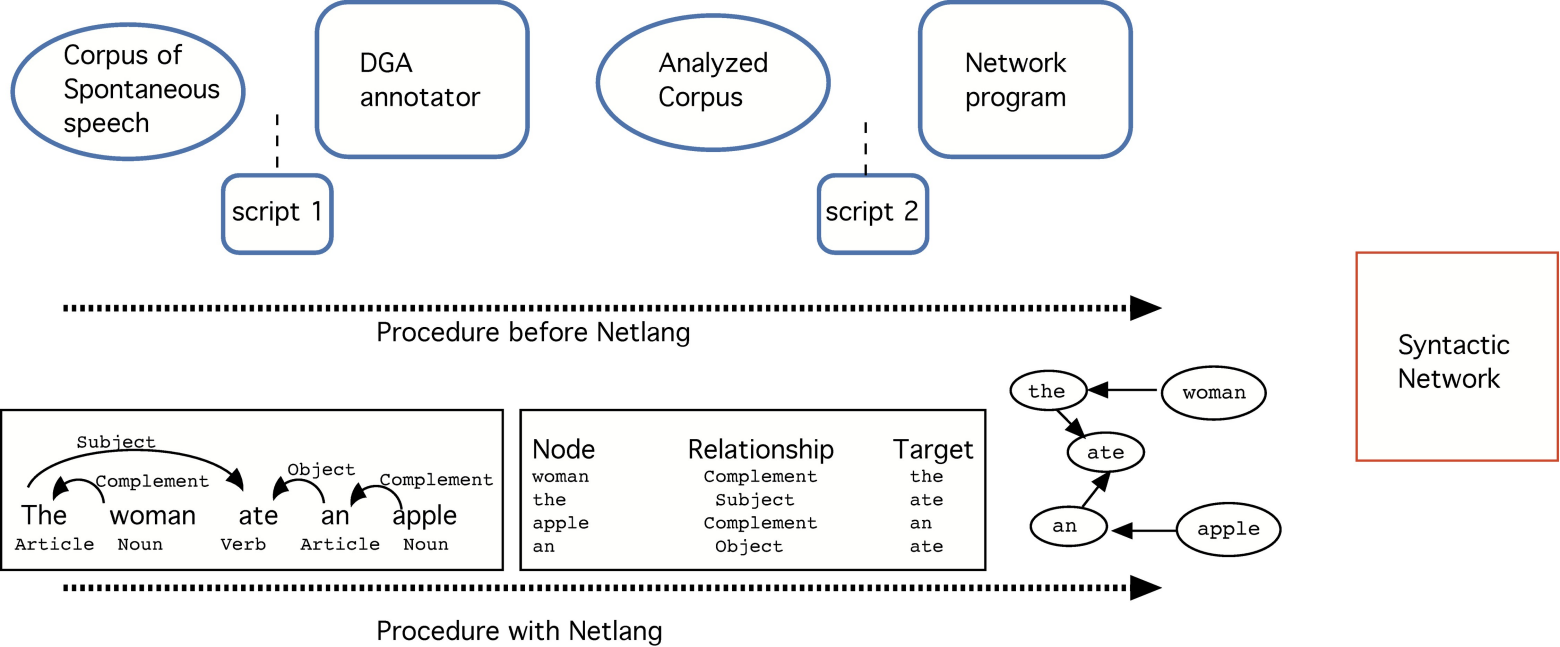
Process followed in the creation of a syntactically annotated network. First, a linguistic corpus was selected. A script was applied in order to get the proper file format. Next, the file was annotated by a linguist who used the DGA annotator. Then, a second script changed the format again into a file readable by a network program. Finally, the network program interpreted the information in terms of a graph of connected elements and was also able to extract statistical information. However, lexical categories (noun, verb, etc) and the labels of the syntactic relationships (object, subject) could not be integrated into the final network.

Finally a network program–like for example *Cytoscape* [38] or *Gephi* [39] is applied to represent the information in the form of a graph and statistically analyse it (Fig 1).

The results obtained from this procedure suggested that there could be commonalities during development between infants of very typologically distant languages, such as Basque (isolate; agglutinative) and Catalan (Romance; non-agglutinative). Moreover, the possibility of tracking the child’s development in typical conditions has raised the question about what happens in atypical conditions. And also what happens in sociolinguistically diverse conditions, like bilingualism or multilingualism. Finally, if this procedure is useful to follow the growth and development of the capacity for language, it might also be helpful for tracking the destruction of that capacity.

**Fig 2:**
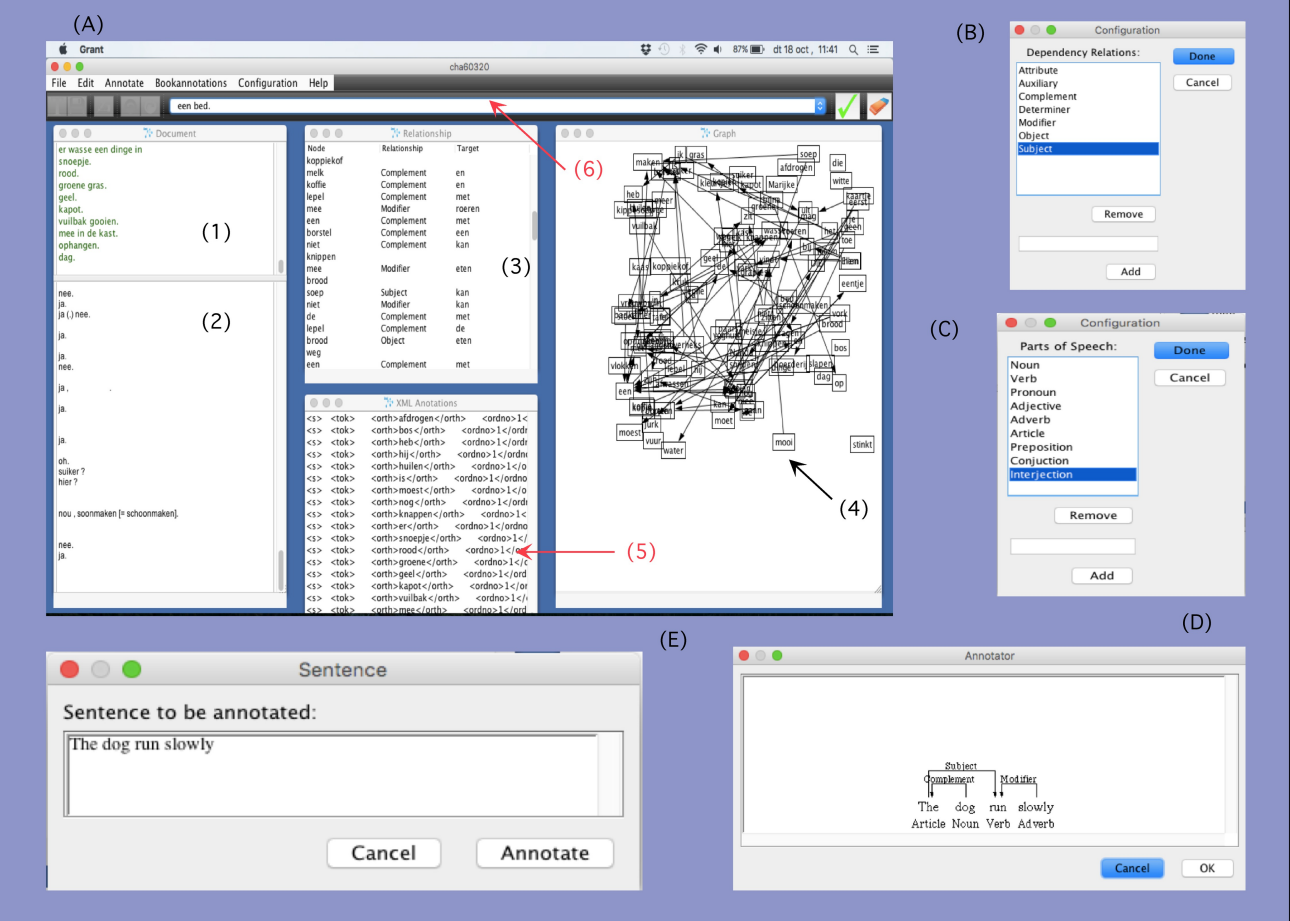
*Netlang* software. (A) Screenshot of *Netlang* software as it is. (A 1) subwindow showing the analyzed text; (A 2) subwindow showing the original text; (A 3) subwindow showing the gathering of the three columns of data; (A 4) window for a first representation of the network; (A 5) internal dependency grammar of *Netlang*; (A 6) in this bare appear all analyzed sentences and the user can select them in order to edit; (B) emergent window from the *Bookannotations* menu that allows to set up the labels for the dependency relations; (C) emergent window from the *Bookannotations* menu for the customization of the labels of word categories; (D) emergent *Netlang* window where syntactic relations have been established between words, by means of arrows which include a syntactic label (e.g., «modifier» or «subject»). Lexical categories have also been identified under the words (e.g., «preposition», «article»). (E) emergent window where the user can check the text to be analyzed and modify it if necessary.

However, the software used until now was rather experimental and sensitive to special characters and some languages were extraordinarily difficult to analyze. The original target was English and when other languages were treated, problems related to special symbols typical of some languages (e.g., the *ñ* in Basque and Spanish, accents in Romance languages, etc) immediately arose. Moreover, the *DGA* program, available online but missing updates for years, presented a big issue regarding the type of platform it was supported on: it had to be Windows (but it did not work in versions later than 2007). With this *DGA* it was also possible in principle to reflect which kind of syntactic relation was (e.g., *subject*, or *object*) and to include lexical labels (e.g., *noun*, *verb*, *adjective*) (see Fig 1), though this information could not be saved once the script 2, i.e. *Xml2Pairs*.*py*, was executed. For these reasons a software has been designed that simplifies the linking process between the linguistic corpus and the network analysis. The main enhancements can be summarized as follows (see also Fig 2):

1. *Netlang* can be executed within a Windows, OSX or Lynux platform.
2. Now it is possible to open any text file and select the proper information of the corpus, and no file transformation is required.
3. It integrates the internal grammar of the *DGA* for the analysis by means of dependency relations in a single application. Including other linguistic theoretical options was very difficult before and now it is even possible to solve a new problem at the very moment on finds it, due to the possibility of editing lexical and syntactic labels and the window for text edition.
  1. Nonetheless, the *Netlang* software is now flexible enough to include the user's preferences in linguistic theory and allows for customization of both lexical categories and syntactic relations.
  2. It is also possible to edit online the target expression. Sometimes a speaker produces a word somehow differently and hence that word has been transcribed differently. These fact will create two different nodes. In some cases it is desirable to unify these expressions (see next section for an example) and now this can be made while analyzing whithin the emergent window.
4. Moreover, a remarkable enhancement is that now *Netlang* includes both lexical information in the nodes and syntactic information into the edges of the network, and can represent this in a single window. Before, the syntactic information of edges was “lost in translation”. This was rather disappointing after a careful syntactic analysis made by hand. Now, this information can be also integrated in the final network.
5. An additional improvement is that *Netlang* includes several options for the output file and hence, it is now possible to export the analyzed text into a format readable by a network program (tested in both *Gephi* and *Cytoscape*). This means that no additional script is now required. Moreover, there are several possibilities for the output.svc, making easy to work with a spreadsheet.

## 3. Methods

In order to test the usefulness of *Netlang*, seven corpora have been analyzed: one corpus of a bilingual child acquiring Spanish and English [40] and then six clinical corpora of a twins study by [41]. The particularity of the latter is that (1) we count with 3 chronologically ordered files per twin (hence, we can track the twins’ linguistic development) and (2) one of the twins suffered a focal lesion, namely a “left intraventricular haemorrhage as a complication of prematurity” [41]. All corpora have been downloaded from CHILDES data base [42].

With the analysis of the bilingual corpus we will explore for instance whether the child mixes Spanish and English. With the clinical twins corpora we will track the development of both children and whether or not the impaired child recovers from the focal lesion.

For the sake of simplicity in this first study the syntactic analysis we have applied includes the following procedural decisions:

1. Words spoken in different ways have been unified online (e.g., *mamma*, *mammma* > “mamma”; *the*, *da*, > “the”). This could be done while analyzing, thanks to the online editing option *Netlang* offers.
2. A series of changes in the set of syntactic relations available in *Netlang* has been adopted according to some theoretical theories:
  1. *Netlang* makes possible to customize the set of lexical or syntactic labels. In this first analysis, the syntactic relation between a verb and its direct object has been labeled *object*. The relation between a preposition and a noun has been labeled *complement*.
    1. We have adopted the so-called *Determiner Phrase hypothesis* or “DP-hypothesis” (*cf*. *i*.*a*. [43, 44, 45, 46, 47, 48]) and hence, within the analysis, a determiner is the *governor* of a noun. Hence, in “an apple”, the noun *apple* syntactically depends on the article *an*. This decision is the consequence of a previous study in which it has been detected that the adoption of the determiner as *governor* of the noun affects the degree of connectivity of hubs [35].
    2. In the bilingual case, the lexical categories were customized introducing just three labels: “English”, “Spanish” and “Proper name”. The intention was to visualize the nodes in different colors, depending on the label they had.

With *Netlang* we have pruned the corpus so that we had just the sentences and utterances spoken by the target child. We have done this by selecting the proper tag, in this case *CHI. Using to the option *edit* >*remove text*, we have also simplified the text by removing symbols that were not necessary, and also the expression “*CHI:”. Then, each utterance has been analyzed, word by word. The output file has been opened in *Cytoscape* and the resultant graph visualized. The giant connected component of the graph (the largest network within the graph) has been analyzed paying attention to the most used features in network science (clustering coefficient, path length, number of nodes, number of edges, average number of edges per node, the network diameter and the ratio of nodes vs. edges).

Finally, thanks to the possibility of extracting information in a.*svc* file, the information of the syntactic analysis was recovered and analyzed in a spreadsheet.

## 4. Results

**Fig 3:**
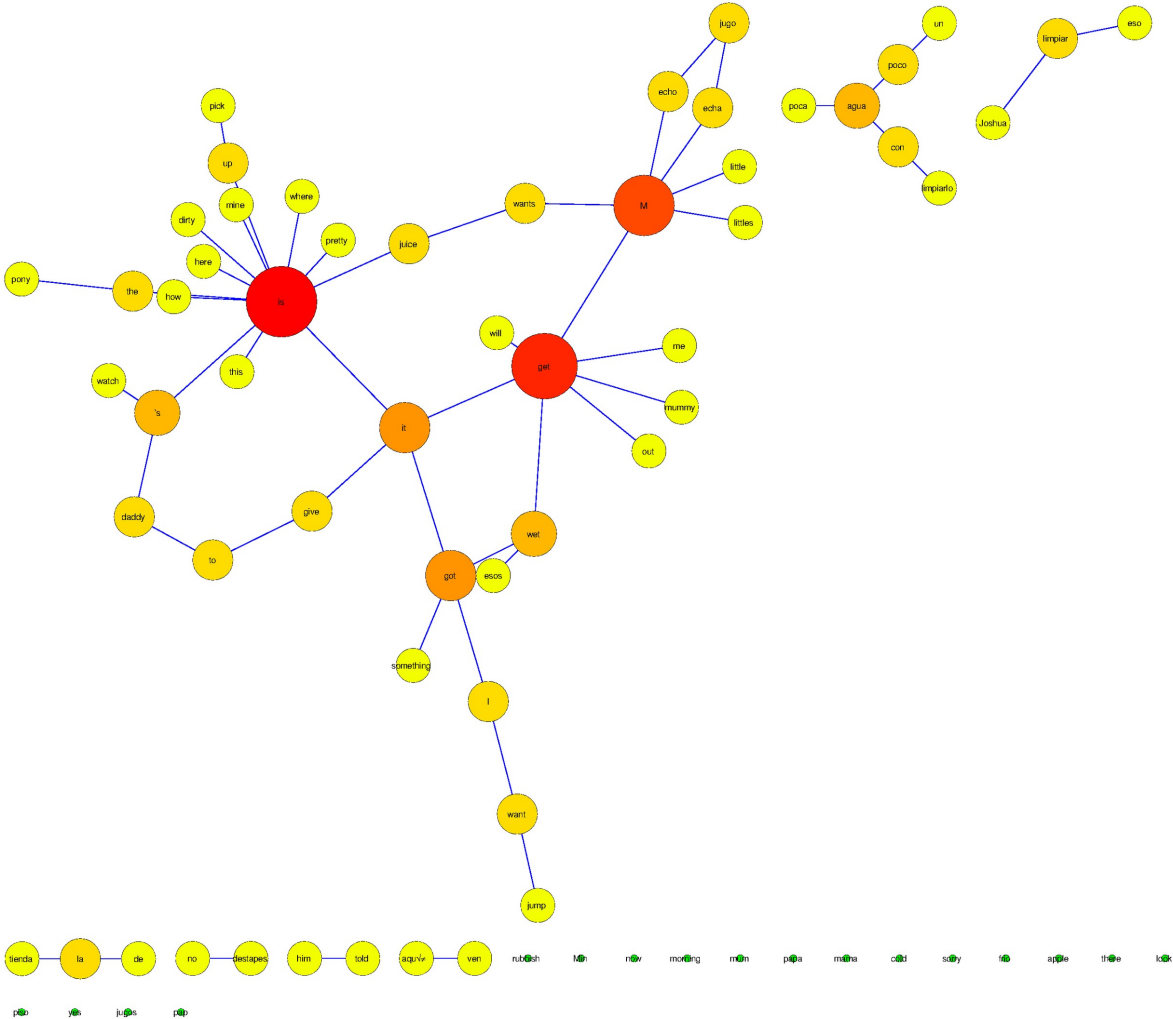
The bilingual network. Lexical categories have been customized in order to reflect whether the word is English or Spanish. An additional third color has been selected for proper names. Syntactic relations are also reflected in the network.

**Fig 4:**
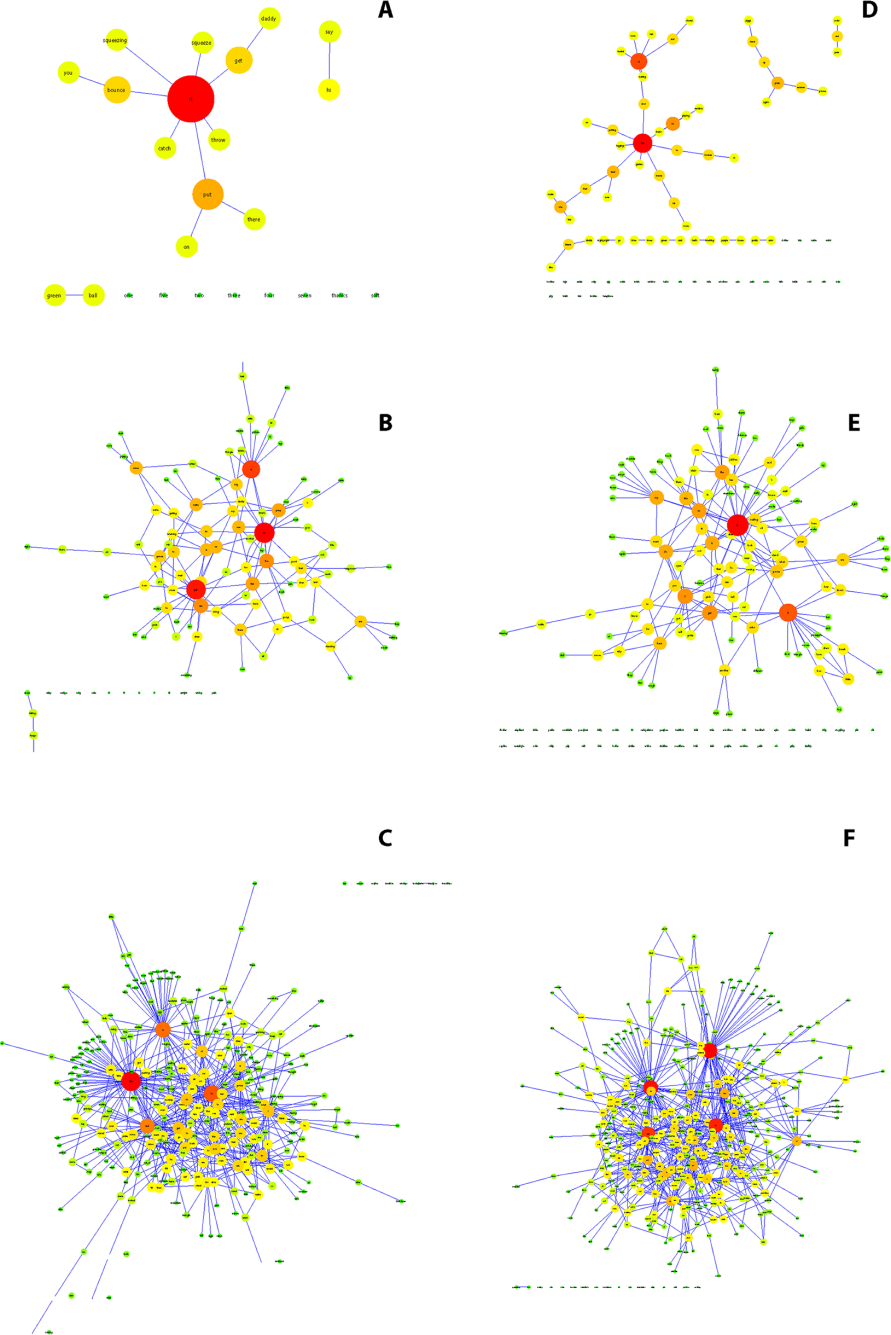
Six networks reflecting the twins’ language ontogeny. In three different periods of their life: at 2 years and 6 months, at 3 years and 1 month and at 7 years. The child MH (files NAM), letters (A), (B) and (C), had a focal lesion.

The networks obtained after the analysis of the linguistic corpora showed that the application of *Netlang* was successful. It was possible to extract the text, to prune the data and the posterior syntactic analysis. The different networks could be screened, showing the words inside nodes and the syntactic labels around the edges (Figs 3 and 4). In the bilingual case, the application of syntactic categories depending on the language, was also successful and the nodes of the graph could be represented in different colors: red for English words, green for Spanish and yellow for proper names.

From the seven corpora we obtained seven graphs and each of them had been statistically analyzed. Information related to the most relevant features of each network is presented in Table 1.

**Table 1:** Main features of the networks as a result from the analysis of corpora by means of *Netlang*. Analysis of the giant connected component of the graph: (*C*) Clustering coefficient, Nodes or number of different words, Edges or number of syntactic links, <*k>* average number of edges per node, *L* or characteristic path length. Age is typically written “*years*;*months*.*days*”, hence the bilingual child is 2 years, 1 month and 20 days old.

	Age	*C*	Nodes	Edges	Ratio n/e	<*k*>	*L*	Diameter
Bilingual children Spanish-English	2;01.20	0	38	41	0.926829	2.158	3.64	8
Child:MH with Focal Lesion (file NAM)	2;6	0	12	11	1.0909	1.833	2.348	4
	3;1	0.015	99	143	0.692307	2.869	3.081	9
	7;0	0.085	287	583	0.492281	3.93	3.387	9
Child: KH unimpaired (file TAK)	2;6	0	29	28	1.035714	1.931	3.837	8
	3;1	0.045	118	174	0.678160	2.915	3.787	9
	7;0	0.067	356	682	0.521994	3.725	3.683	8

The twins corpora have made possible a different analysis of that case study by adopting the viewpoint of complexity. It has been possible to see the differences between twins at the age of 2 years and 6 months: the unimpaired twin almost produced two times more different words and syntactic links. 7 months later the twin with the focal lesion seems to make progress (related it to brain plasticity [41]). 4 years later both twins produce a considerable number of different words and syntactic links, but the unimpaired twin has produced almost 100 words and syntactic links more than her sibling. Nevertheless, Feldman and collaborators noted that the twin with focal lesion was tired that day and that this fact probably had affected the linguistic production during the session. The final network (at 7 years) can be considered as a small-world network, since it has a high clustering coefficient along with a low path length. Moreover, the final network has a ratio nodes/words vs. edges/syntactic links near to 0.5, i.e. for each word the child produces two syntactic links.

The results of our networks coincide with the original work and have been able to track the development of these two children by providing a new look to the same data.

A further improvement of this kind of analysis is that *Netlang* allows the data to be exported in.svc format. Hence we exported the data and observed the frequency of syntactic relations between words (Table 2). In the bilingual case this is irrelevant because the study was not longitudinal nor it aimed this purpose. However, in the twins study this allows us to see which syntactic relations are more frequent, which are absent–if any (especially relevant in the clinical condition)–and whether or not an absent syntactic relation does appear again (this could suggest a recovery).

**Table 2:** Number of syntactic relations classified by age and label, recovered from each whole graph (hence, including the giant connected component and other smaller networks).

Child: KH (file TAK)			
Age	2;6	3;1	7;0
Complement	18	82	329
Modifier	18	51	246
Object	8	130	94
Subject	1	57	152
Attribute	0	23	28
Child: MH (file NAM) focal lesion			
Age	2;6	3;1	7;0
Complement	0	85	340
Modifier	3	59	205
Object	9	30	75
Subject	2	37	172
Attribute	0	16	21

We have also paid attention to the most connected words, the so-called *hubs* of the networks. Hubs are highly connected nodes that play an important role in scale-free networks. A longitudinal study can reveal the progress of the words’ connectivity and how some words begin with a low connectivity and at some point during ontogeny, these words gain many connections. In previous works the so-called functional words stand out regarding the number of direct links or edges. In Table 3 lexical hubs and their number of directed edges have been recovered.

**Table 3:** Hubs (or highly connected words) of the networks in three temporal periods, at 2.6, 3.1 and 7 years of the child’s life.

Child	Age	Hubs	Number of directed edges	Child	Age	Hubs	Number of directed edges
MH	2.6	it	7	KH	2.6	the	8
		put	3			a	6
	3.1	put	14		3.1	is	23
		is	15			a	16
		a	12			get	11
		the	8			I	11
		can	8			the	10
						on	10
	7	the	74			it	9
		is	51			do	9
		a	44			this	8
		and	39				
		on	21		7	a	57
		get	20			the	54
		I	20			is	50
		of	19			and	46
		do	18			are	20
		you	17			you	20
		can	15			was	20
		are	15			get	19
		in	15			of	18
		gonna	14			to	17
		it	14			he	14
		what	14			make	14
		put	14			I	14
		to	14			some	13
		use	13			it	13
		this	12			that	12
		was	12			one	12
		some	10			on	12
		all	10			for	11
						in	11
						go	10
						will	10
						this	10
						like	10

## Conclusions

In the present work a new computational has been applied to the syntactic analysis of linguistic corpora by means of complex networks. Results show that the procedure has improved considerably by reducing the number of programs and scripts, and solving a number of problems, some of them related to the storage of morpho-syntactic information and some others related to the language typology. Both lexical and syntactic information can be saved and reflected in the network: a node contains the original word, but it can also contain an additional feature related to the lexical category. In the bilingual case, we have gone further and have customized the categories in order to represent whether a word was either Spanish or English. Other possibilities are available, depending on the targets of the study. It is true that *Netlang* does not do the analysis for the linguist but this feature makes the software useful for the analysis of any language regardless of its linguistic typology (for the moment, languages using alphabets or codifying systems different from the Greco-Latin alphabet still need to be transcribed).

With the help of *Netlang* it has been possible to track the evolution of a pair of twins, one typically developing and the other atypically developing due to a left intraventricular haemorrhage. The linguistic development of these children could be analyzed with *Netlang* and then the resultant analysis could satisfactorily reflected in the form of a graph. The statistical analysis of the networks shed some new light, showing that this way of analysis of the corpora can be complemented with new information from other kind of analyses. This information cannot be seen by the unaided eye and now the procedure for its recovering has been eased with the new software.

Additionally, it is easy to see the progress of the impaired child. In the case of the impaired child, it is particularly interesting the absence of the determiners “the” and “a” as hubs in the first period, since these words had been detected as crucial hubs in two typically developing, English speaking children by [25]. Nonetheless, at 3;1 years the impaired child seems to have recovered in many regards though the differences between siblings are still evident. In the third file yet at 7 years the impaired child seems to have recovered substantially. Both children produce similar results, and in both cases the network is a small-world network with a similar, interesting ratio of words vs. syntactic links or edges (Table 1). During the first and second period the differences between twins are evident. It is in the third period when MH seems to have evolved quite positively. At least with regard to syntactic constructions, number of different words and number of different syntactic links and network structure, numbers are comparable and in some cases even superior to the unimpaired sibling (Table 2).

In conclusion, studies on language acquisition in both typical and atypical conditions and on bilingualism or multilingualism, have gained a new tool that can complement other kinds of analysis. *Netlang* software could be enhanced in several ways in the future. Since it is central to the enterprise *Netlang*. *Complex Networks and Language*, future developments and updates will be announced on https://neurolang.wordpress.com. One of the ways that we envisage could be enhanced would be by including more statistical information related to the edges and the nodes, for example, frequency: how many times a word has been said, or how many times a particular phrase has been produced. Since the software is open-source, modifications can be made by anyone.
